# Formation and Evolution of Soot in Ethylene Inverse Diffusion Flames in Ozone Atmosphere

**DOI:** 10.3390/nano13050816

**Published:** 2023-02-22

**Authors:** Yaoyao Ying, Dong Liu

**Affiliations:** 1 MIIT Key Laboratory of Thermal Control of Electronic Equipment, School of Energy and Power Engineering, Nanjing University of Science and Technology, Nanjing 210094, China; 2 Advanced Combustion Laboratory, School of Energy and Power Engineering, Nanjing University of Science and Technology, Nanjing 210094, China

**Keywords:** inverse diffusion flame, ozone, soot, nanostructure, oxidation reactivity

## Abstract

Ozone is a prospective additive for enhancing and controlling combustion under lean or very lean conditions, and reduces NOx and particulate matter emissions simultaneously. Typically, in studying the effects of ozone on combustion pollutants, the focus is on the final yield of pollutants, while its detailed effects on the soot formation process remain unknown. Here, the formation and evolution profiles of soot containing morphology and nanostructures in ethylene inverse diffusion flames with different ozone concentration additions were experimentally studied. The surface chemistry and oxidation reactivity of soot particles were also compared. The soot samples were collected by a combination of the thermophoretic sampling method and deposition sampling method. High-resolution transmission electron microscopy analysis, X-ray photoelectron spectroscopy and thermogravimetric analysis were applied to obtain the soot characteristics. The results showed that soot particles experienced inception, surface growth, and agglomeration in the ethylene inverse diffusion flame within a flame axial direction. The soot formation and agglomeration were slightly advanced since the ozone decomposition contributed to promoting the production of free radicals and active substances in the ozone added flames. The diameter of primary particles in the flame with ozone addition was larger. With the increase of ozone concentration, the content of soot surface oxygen increased and the ratio of sp^2^/sp^3^ decreased. Furthermore, the addition of ozone increased the volatile content of soot particles and improved soot oxidation reactivity.

## 1. Introduction

To reduce the emissions of NOx, modern gas turbines usually operate under ultra-lean conditions near the flame extinction limit, revealing that reliable technologies to improve flame stability are essential [1]. As another negative pollutant, soot is also widely investigated by preventing or reducing the production of soot through various methods [2,3]. Plasma-assisted combustion shows broad application prospects in improving combustion and controlling flames [4,5,6,7]; it can maintain normal operation under lean or very lean conditions and reduce NOx and particulate matter emissions simultaneously [4,8]. Plasma-flame coupling can generate free electrons, ions, active radicals, excited molecules, etc., which accelerate the oxidation process of fuel, shorten the ignition delay time, and enhance the flame propagation speed [9,10]. However, free electrons, ions, and active radicals have short lifetimes and are difficult to apply practically. Therefore, excited molecules with longer lifetimes are more practical for existing combustion devices. Ozone (O_3_) is considered one of the most promising active molecules, with a long lifetime and strong oxidizing properties to improve combustion stability [11,12].

Nishida and Tachibana [13] studied the effect of ozone addition on the ignition of premix combustion of natural gas in a homogeneous charge compression ignition (HCCI) engine, and found that the ignition timing can be controlled by altering the ozone concentration, and its effect on the ignition angle was almost the same as that of O radical. Yamada et al. [14] conducted a composition analysis of ozone additive effects on ignition timing in the HCCI engine of dimethyl ether. The results showed that ozone could promote the ignition timing and increase heat release in the flame, and that ozone contributed by decomposing into O + O_2_ at the beginning of combustion. Halter et al. [15] studied the effect of ozone on the burning velocity of methane/air premixed flames at atmospheric pressure and room temperature with experimental and numerical methods. The result showed that when some of the oxygen molecules were converted to ozone, the flame burning velocities increased by about 3–8% with 5 g/Nm^3^ ozone concentration in air. Ombrello et al. [16] investigated the thermal and kinetic effects of ozone on flame propagation in a laminar non-premixed C_3_H_8_ flame by experimental and numerical methods. The experimental result showed that the flame propagation speed was enhanced by 8% when the ozone concentration in the oxidizer stream was 1260 ppm. The enhancement in combustion and flame propagation speed could be attributed to the ozone decomposition in the preheating stage. Wang et al. [17] investigated the effect of ozone on the burning velocity in premixed methane flames experimentally and numerically, and found that a noticeable enhancement of burning velocity was observed with ozone addition, which could be attributed to the extra O radicals in ozone atmosphere advancing the chain-branching reactions. This phenomenon could also be observed when ozone was added to the combustion of ethylene, H_2_/CO, n-heptane, iso-octane and other fuels [18,19,20]. Vu et al. [21] carried out a study focusing on the influence of ozone on blowoff characters by using premixed methane and propane Bunsen flames, and found that ozone could effectively enhance the blowoff velocity and extend the flammability limits of fuel mixtures. Zhang et al. [22] numerically investigated the effect of ozone on the flammability limit and near-limit combustion of H_2_/CO flames with different dilution gases. They also found that with ozone addition, the flammability limit expanded and the laminar flame velocity increased, and the enhancement effect on the flame velocity was more significant under near-limit conditions.

The effects of ozone on combustion pollutants have also been studied. Tachibana et al. [23] investigated the effect of ozone addition on combustion characteristics in compression ignition engines and found that CO, hydrocarbons, and soot particulates all decreased. Wilk and Magdziarz [24] found similar results, that ozone additions to natural gas combustion led to a decrease in CO and hydrocarbons’ concentrations in a modified Mecker burner. In addition, ozone has also been applied in processing exhaust gas. Mok and Lee [25] proposed a two-step process to simultaneously remove NO*_x_* and SO_2_ by injecting ozone, and the result showed that the removal efficiency of NO*_x_* and SO_2_ was about 95% and 100%, respectively. Wang et al. [26] further investigated the simultaneous removal of NO*_x_*, SO_2_, and Hg by ozone injection in a quartz flow reactor, and they found that the removal efficiency of NO and Hg gradually improved with increasing ozone concentration. A similar result was found by Sun et al. [27] when simultaneously removing NO*_x_* and SO_2_ using ozone, that NO*_x_* removal efficiency enhanced with increased ozone addition. Holder et al. [28] investigated the physical and chemical properties and cellular response of combustion-generated particles with oxidants of ozone and nitrogen dioxide, and the results showed that soot reacted with ozone had different chemical properties and was more toxic. Browne et al. [29] used an aerosol flow reactor to obtain soot aging features with heterogeneous oxidation by OH and ozone, and found that heterogeneous reactions with OH and ozone were effective on the oxidation of soot particles, especially the aliphatic organic species on soot. Daly and Horn [30] compared the heterogeneous reaction chemistry of soot produced from toluene, kerosene, and diesel with ozone oxidizing, and the results showed that ozonized soot presented different reactivities due to the ratio of organic carbon and elemental carbon.

Previous studies have investigated the effects of ozone on flame ignition delay time, laminar flame velocity, flame stability, and combustion pollutants both experimentally and numerically. The addition of ozone improves combustion and laminar flame speed, shortens ignition delay time, expands flame flammability limit, and enhances combustion efficiency. The oxidizing reactions and chemical properties are affected by the ozone atmosphere during the course of soot oxidation. Therefore, it could be speculated that ozone addition in flame influences the generation of soot in combustion. However, these studies have not explored the particles produced with ozone during the combustion process, and the effects of ozone on soot properties in flame remain unknown.

Thus, the present study aims to obtain the evolution profiles of soot in ethylene inverse diffusion flames in ozone atmosphere, including physical and chemical characteristics. The soot particles are collected by local and global sampling methods as in our previous studies [31,32]. The profiles of soot morphology and nanostructure are obtained by high-resolution transmission electron microscopy (HRTEM) using the local sampling method—a thermophoretic sampling technique—to gather soot particles along the flame axial direction. The global method—a quartz plate sampling system—was applied to collect soot in the post-flame region for information on the elemental composition and oxidation reactivity of the soot with different ozone concentrations analyzed by X-ray photoelectron spectroscopy (XPS) and thermogravimetric analysis (TGA).

## 2. Experimental Setup and Procedures

**Figure 1:**
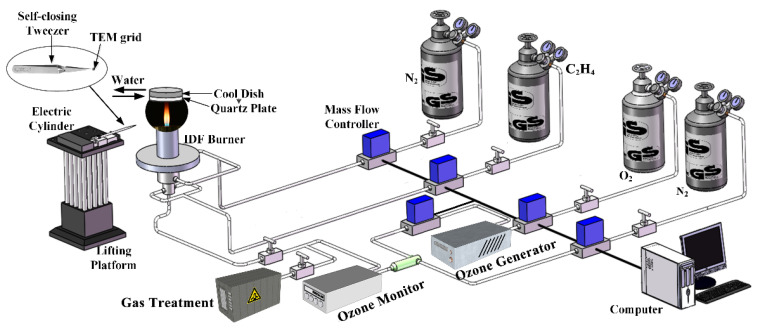
Schematic diagram of experimental set up.

An inverse diffusion flame (IDF) burner, the same as in the previous works [31,32,33,34], is used in this study. The base fuel C_2_H_4_ is supplied by the intermediate tube, and the shield gas N_2_ is provided in the outer tube. To generate an O_3_ atmosphere, an ozone generator is connected to the O_2_ gas pipeline, and then part of the O_2_ is converted into O_3_. The partially O_3_ and O_2_ mixture gases are supplied to the central tube of the IDF as the oxidizer. The O_3_ concentration is measured online by an ozone detector, which is connected to the outlet of the mixing device of oxidizer gas from the ozone generator and diluent gas N_2_. The detailed experimental system is shown in Figure 1. The soot forms on the outside of the flame and moves upward through the cooler regions of the fuel stream.

During the experiments, the flow rate of O_2_ gas in the flames is fixed. The concentration of ozone is changed by adjusting the discharge power of the ozone generator. According to previous studies on ozone’s effects on combustion characteristics with different concentrations [1,15,16,17,18,20,21] and to ensure the stable operation of the ozone generator in the study, the ozone concentrations added in this paper are 5.4 ± 0.3 mg/L and 10.9 ± 0.5 mg/L, which are abbreviated as O5 and O10 to the corresponding flame conditions, respectively. The flame condition is abbreviated as O0 without ozone. The detailed flame conditions are listed in Table 1.

The flame temperatures were measured by the rapid insertion method with a B-type thermocouple [32,33]. To ensure measurement accuracy, the measurements were repeated at least three times and radiant heat losses were corrected [35]. The uncertainty of the temperature measurement was within ±50 K. The soot samples were captured by a combination of local and global sampling methods [31,32]. Briefly, the local sampling method—the thermophoretic sampling technique—was used to capture the soot directly by TEM grid along the flame boundary line at various heights above the burner (HAB) at 4, 10, 20, 30, and 40 mm. Then, the TEM images of the soot were obtained by a Tecnai G2 F30 S-TWIN transmission electron microscopy (FEI, Hillsboro, OR, USA) to analyze the evolution profiles containing morphology and nanostructure. The global sampling method—a quartz plate sampling system—was applied with a diameter of 95 mm plate to collect soot samples in the post-flame region at HAB = 40 mm for analysis of surface chemical properties by a Thermo Scientific Escalab 250 Xi instrument (ThermoFisher, Waltham, MA, USA). Moreover, the soot oxidation reactivity was tested by an STA 449 F3 Jupiter thermogravimetric analyzer (NETZSCH, Selb, Germany) at an isothermal temperature of 500 °C. Detailed characterization analysis methods could be found in the previous studies [31,33].

## 3. Results and Discussion

### 3.1. Flame Typical Features

**Figure 2:**
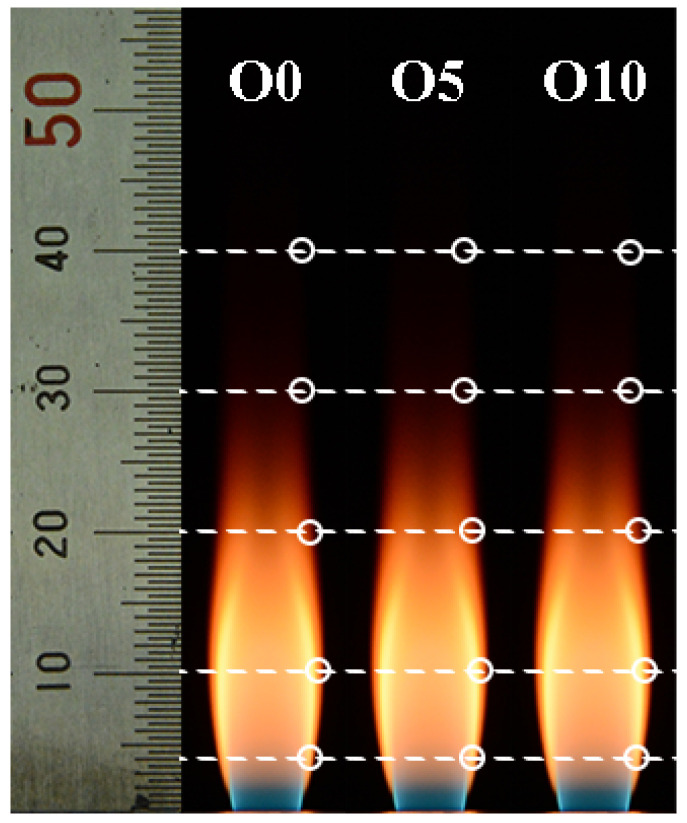
Flame images at different ozone concentrations. The circles display the thermophoretic sampling locations along flame boundary line.

The flame images with different ozone concentrations are shown in Figure 2. The small circles in the figure represent the specific locations of thermophoretic sampling at HAB = 4, 10, 20, 30, and 40 mm along the flame boundary line. Whether O_3_ is added or not, the flame is very bright. The naked eye cannot directly observe the flame variation when the ozone concentration changes.

**Figure 3:**
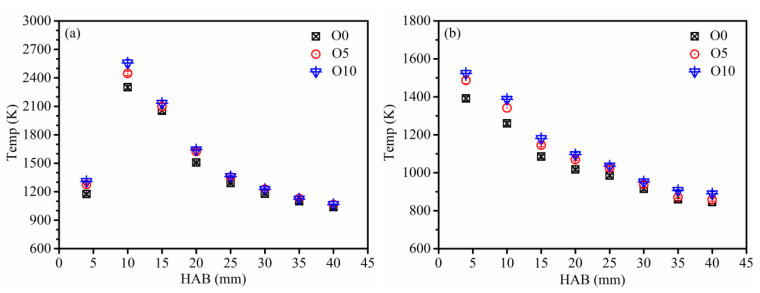
Measured temperature distributions of flame at (**a**) center line, (**b**) boundary line at different HAB with different ozone concentrations.

The flame temperature distribution at different heights of the flame center line and boundary line (including the thermophoretic sampling locations) is presented in Figure 3. With the increase in ozone concentration in the flame, the temperature at the same position on the flame center line increases, which is consistent with the results of previous studies [15,17]. At the lower flame position of HAB ≤ 15 mm, which is the main reaction region, the effect of ozone on temperature variation is more significant. On the flame center line, at HAB = 10 mm, the maximum flame temperature of O0, O5, and O10 are 2300 K, 2446 K, and 2556 K, respectively. With the increase in HAB, the flame temperature decreases gradually, and the difference in flame temperature reduces under different flame conditions. On the flame boundary line, the flame temperature decreases with the increase in HAB, and its changing trend is consistent with the temperature variation tendency in previous studies.

**Figure 4:**
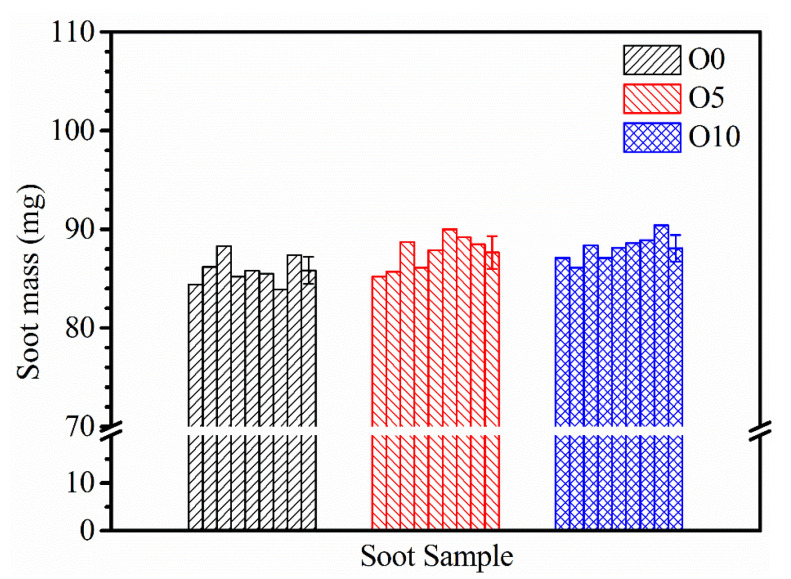
Mass distribution of soot samples deposited at HAB = 40 mm with different ozone concentrations.

Figure 4 shows the mass distribution of soot particles collected by quartz glass deposition at HAB = 40 mm with different ozone concentrations. It can be seen that the difference in the mass of soot samples obtained by single deposition sampling is not obvious under different conditions. With the increase in ozone concentration, soot production increases slightly. The average mass value and the error bar of soot samples with each ozone concentration are presented on the right of the column image of each working condition. The average mass of soot collected in O0, O5, and O10 flames was 85.8 ± 1.37, 87.7 ± 1.66, and 88.1 ± 1.24 mg, respectively. There is no significant change in soot generation when the concentration of ozone in flame increases.

### 3.2. Soot Evolution Profiles

**Figure 5:**
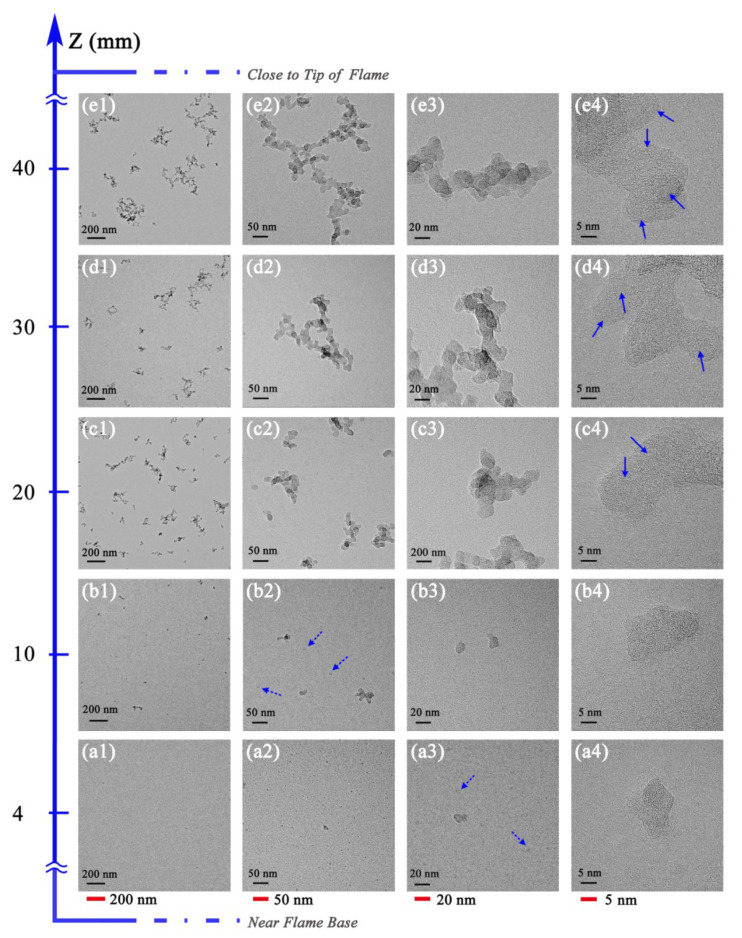
Morphology and nanostructure profiles of soot in ethylene inverse diffusion flame at different HAB without ozone addition, (**a1**,**b1**,**c1**,**d1**,**e1**) at a resolution of 200 nm, (**a2**,**b2**,**c2**,**d2**,**e2**) at a resolution of 50 nm, (**a3**,**b3**,**c3**,**d3**,**e3**) at a resolution of 20 nm, (**a4**,**b4**,**c4**,**d4**,**e4**) at a resolution of 5 nm.

Figure 5 presents the evolution profiles containing information on the morphology and nanostructure of soot particles at different HAB without ozone addition, showing the different stages of soot in the formation process, including soot inception in a low region followed by particle growth and agglomeration, and subsequent soot carbonization at higher positions. Lower magnification TEM images provide the particle density information of soot aggregates at different flame heights (Figure 5(a1,b1,c1,d1,e1)). The TEM images with higher magnification (Figure 5(a2,b2,c2,d2,e2)) can be used to measure the particle size of primary soot particles. It can be seen from the TEM images that the size of primary particles, the degree of agglomeration, and the density of aggregates are strongly dependent on the flame position.

In the upper part of the flame, the soot particles present long chains or large clusters, and the aggregates are more branched. While in the lower part of the flame, the soot particles are mainly composed of individual particles, and aggregates are governed by a smaller number of primary particles. The TEM image of the soot gathered from the lowest sampling position (HAB = 4 mm) (Figure 5(a2)) indicates the existence mainly of single particles; the particles have just started to form and are very rare and difficult to find. Such individual particles are often referred to as young soot in the literature [36,37,38]; these particles are relatively transparent and have low contrast in TEM images (Figure 5(a1,a2,a3,a4)). It is generally believed that these low-contrast particles are associated with the presence of aliphatic hydrocarbons [39,40] because they have a higher H/C ratio than aromatic hydrocarbons. The higher the H/C ratio is, the lower the degree of solidification of the particles.

In the flame at HAB ≤ 10 mm, there are singlet particles with higher transparency and unclear shapes and boundaries (indicated by dotted arrows). Previous studies [41,42,43,44] have described that soot precursors are transparent to visible light and present in the lower temperature fuel-rich regions; in the literature, such singlet particles usually show the liquid-like features. The structures with such characteristics that are present in the lower part of the flame are precursors to the formation of soot particles [45,46]. Then, from HAB = 10 mm to 20 mm, the particle diameter increases due to the coalescence and surface growth, as soot precursors move to the upper region of the flame. The agglomeration degree of soot particles increases rapidly from HAB = 20 mm (Figure 5(c1)). At this height, particle growth and agglomeration prevail, and the particles are formed by short aggregates with irregular shapes and high density. As the particles move higher to HAB = 30 mm to 40 mm, the agglomeration and soot carbonization are predominant in the upper regions [36]; subsequently, the soot particles are governed by long and branch aggregates.

The HRTEM images in Figure 5(a4,b4,c4,d4,e4) show the nanostructure of soot at different evolution stages in the flame. At the lower position, soot particles have just formed with disordered internal structures and short fringes, and the boundary between the particle surface and TEM carbon film is fuzzy. Meanwhile, the singlet particles tend to form larger particles, and there is no obvious edge between particles. As the particles move upward toward the flame tip, the internal structure changes significantly. With the increasing sampling height, soot particles grow up and agglomerate continuously, and the carbonization degree increases. In the upper flame region, fullerene-like nanostructures (indicated by arrows in solid lines) appear inside the particles, and the large or small shells increase. The fringes with longer lengths are more distinguishable, and the arrangement becomes more organized. The higher the sampling height of soot is, the more ordered the degree of fringes.

**Figure 6:**
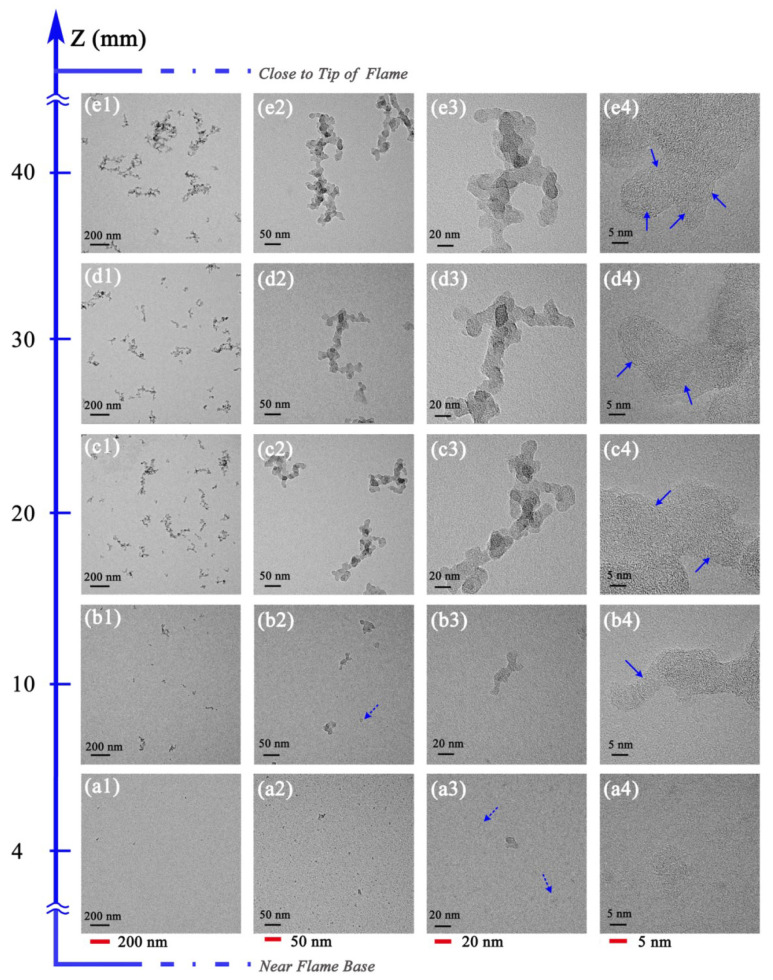
Morphology and nanostructure profiles of soot in ethylene inverse diffusion flame at different HAB with 5.4 mg/L ozone addition, (**a1**,**b1**,**c1**,**d1**,**e1**) at a resolution of 200 nm, (**a2**,**b2**,**c2**,**d2**,**e2**) at a resolution of 50 nm, (**a3**,**b3**,**c3**,**d3**,**e3**) at a resolution of 20 nm, (**a4**,**b4**,**c4**,**d4**,**e4**) at a resolution of 5 nm.

Figure 6 shows the evolution process of the morphology and nanostructure of soot in O5 flame. The variation tendency of soot characteristics is similar to that of soot in the O0 flame. In TEM images with lower magnification, the soot particles are just starting to form and are hard to find at HAB = 4 mm. At HAB = 10 mm, the particles are continuously produced and the number of particles increases. The soot particulates are governed by singlet particles and aggregates with a small number of primary particles in this flame region. There are also some relatively transparent irregular liquid-like substances (indicated by dotted arrows), which are precursors to the formation of solid soot particles. The soot inception process begins in the lower regions. With the increase in sampling height from HAB = 10 mm to 20 mm, the diameter of the primary particle and the density of aggregates increase accordingly due to surface growth and collisions.

When the sampling height increases from HAB = 30 mm to 40 mm, the soot particles are mainly composed of long or large clusters with more branches, as shown in Figure 6(e1). In the upper regions, the soot particles are undergoing the processes of soot agglomeration and carbonization. It can be seen from the TEM images of lower magnification that, compared with O0 flame, the number and distribution density of soot particles in O5 flame are slightly increased, which is consistent with the average mass of soot obtained by deposition sampling. The growth of soot particles in the O5 flame occurs slightly earlier than that in the O0 flame at the same sampling height. This is because the decomposition of ozone produces active O radicals, which accelerates the combustion reaction and subsequently advances the soot formation process [15,17].

The nanostructure characteristics of soot at different HAB in O5 flame are shown in Figure 6(a4,b4,c4,d4,e4). At HAB = 4 mm, the inner carbon layer of soot particles is disordered, and the boundary between the particle surface and TEM carbon film is not clear. With the increase in sampling height, the contrast of the particle surface is enhanced and the soot nanostructure changes accordingly, so that the arrangement of the carbon layers with the particles becomes more organized, and a clear fullerene-like structure appears (illustrated by solid arrows). In the upper area of the flame, the fullerene-like structures in the soot particles are more prominent. The fringes with relatively long lengths are arranged around to form shells of different sizes. The nanostructure arrangement and the fringe length and tortuosity strongly depend on the sampling height in the flame.

**Figure 7:**
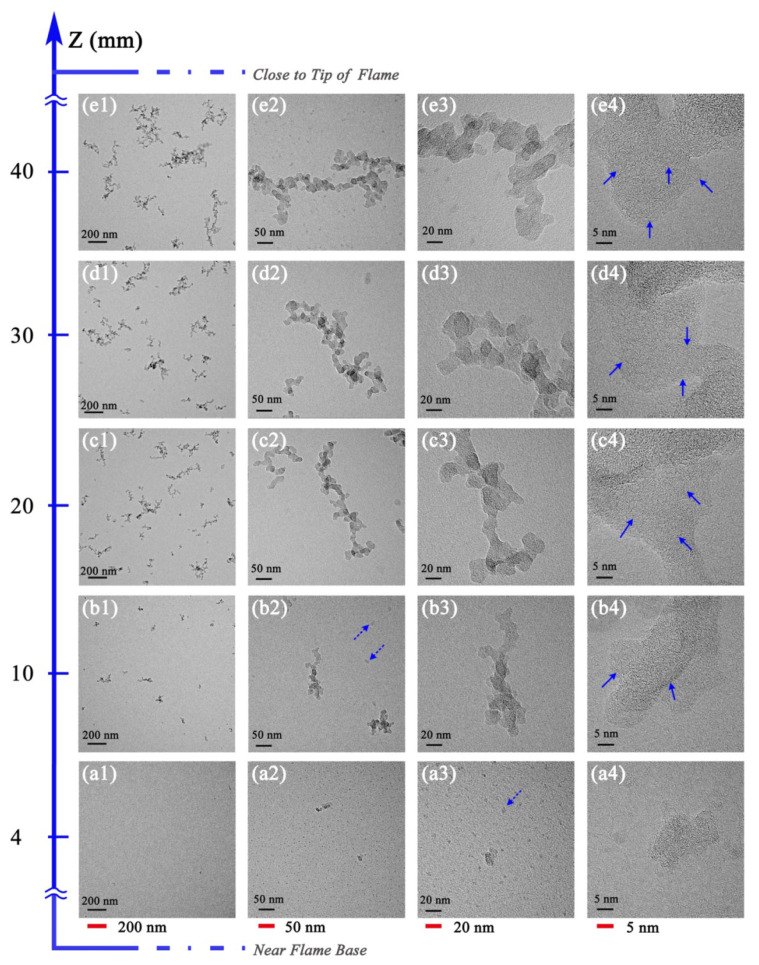
Morphology and nanostructure profiles of soot in ethylene inverse diffusion flame at different HAB with 10.9 mg/L ozone addition, (**a1**,**b1**,**c1**,**d1**,**e1**) at a resolution of 200 nm, (**a2**,**b2**,**c2**,**d2**,**e2**) at a resolution of 50 nm, (**a3**,**b3**,**c3**,**d3**,**e3**) at a resolution of 20 nm, (**a4**,**b4**,**c4**,**d4**,**e4**) at a resolution of 5 nm.

Figure 7 shows the evolution profiles of soot morphology and nanostructure in the O10 flame; the variation law of its characteristics is basically consistent with that of soot particles in O0 and O5 flames, presenting the main soot formation stages of soot inception, particle growth and agglomeration, and soot carbonization.

With lower magnification at HAB = 4 mm, the visible particulates are rare, as the particles are just beginning to form. In this flame region, liquid-like materials (indicated by dotted arrows) with irregular shapes and high transparency coexist with solidified soot particles. The presence of these irregular-shaped features shows the soot inception course. At HAB = 10 mm, there are still singlet particles, while a small number of aggregates also appear, composed of several primary particles. The small aggregates appear earlier than that in O0 and O5 flames, illustrating a promotion in soot particle growth with higher ozone concentration. The effect of ozone addition on the facilitation of soot formation can be understood by considering the role of ozone decomposition. Ozone decomposes through the reaction O_3_ + (M) = O + O_2_ + (M) and releases O atoms [21,47]. It is widely accepted that H-abstraction-C_2_H_2_-addition (HACA) is important in soot formation [48,49,50]. The atomic O accelerates the H abstraction reaction rate of the fuel C_2_H_4_, and C_2_H_4_, then converts to important intermediates through two possible pathways: (1) C_2_H_4_ + O = CH_3_ + HCO, and (2) C_2_H_4_ + H(+M) = C_2_H_5_ (+M) [18]. The consumption pathways of C_2_H_4_ lead to subsequent reactions relating to soot inception. Thus, the soot formation process is promoted in the flame with a higher ozone concentration. The diameter of the particles increases due to the surface growth from HAB = 4 mm to 10 mm.

When the sampling height was increased from HAB = 10 mm to 20 mm, more chains of clustered soot aggregates appeared. In this region, the dominant process is soot particle growth and agglomeration. In the middle regions of the flame, the primary particle size increases because of surface growth and PAH condensation, and the agglomerates grow due to a cluster–cluster aggregation (CCA) by collisional growth [51], which subsequently results in a reduction in singlet particulates and an increase in aggregate clusters. As the sampling height continued to rise (HAB = 30 to 40 mm), the soot surface growth gradually slows down, and the collisional agglomeration and soot carbonization becomes predominant, forming a large number of soot aggregates composed of dozens or hundreds of primary particles. The aggregates have an obvious branching structure and contain more basic particulates. Compared with O0 and O5 flames, more soot is generated in the O10 flame with the increase in ozone concentration.

The nanostructure images of soot at different formation stages in O10 shown in Figure 7(a4,b4,c4,d4,e4) illustrate that all samples present recognizable crystalline carbon layers of different sizes. The arrangement of the carbon layers at HAB = 4 mm is chaotic and without clear rules, since the particles have only just formed. At HAB ≥ 10 mm, soot particles develop with the increasing sampling height, and the longer carbon layers are more ordered, with the presence of a fullerene-like nanostructure (indicated by solid arrows). The higher the sampling height is, the more prevalent the fullerene-like structure and the more ordered the arrangement.

**Figure 8:**
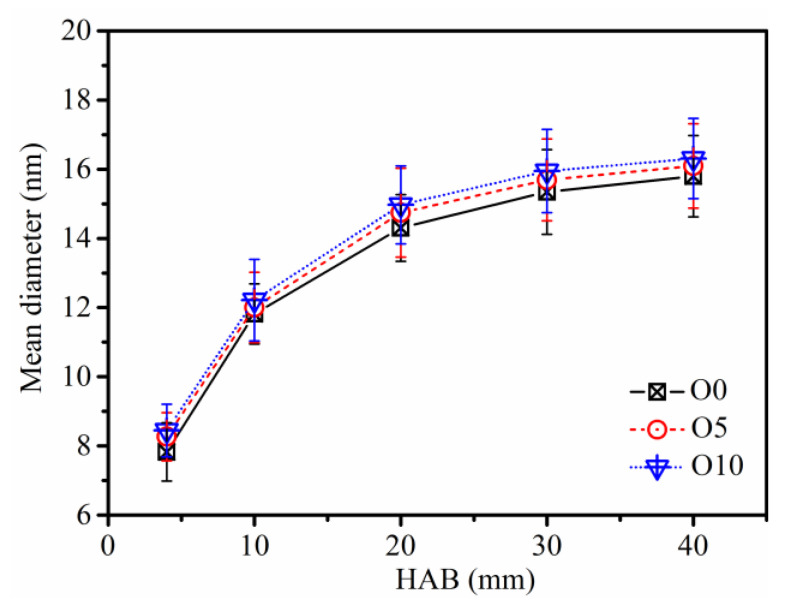
Average diameter of primary particles with different ozone concentrations at different HAB.

The primary particle diameter increases gradually due to surface growth and PAH condensation as the particles move from the bottom to the tip of the flame. A quantitative analysis of the primary particle diameter in the flames with different ozone concentrations is shown in Figure 8. At HAB = 4 mm, the size of the primary particle is in the range of 7.8 to 8.4 nm, since the particles are just starting to form and the presence of singlet particles referred to as young soot reveals the particle inception process.

With the increase of sampling height, the diameter of particles increases due to surface growth. From HAB = 4 mm to HAB = 20 mm, the particle diameter increases rapidly due to the dominant surface growth and agglomeration processes. The particle size growth trend slows down from HAB = 20 mm to HAB = 40 mm because in this region, the growth of particle surface gradually ceases, while the agglomeration and carbonization of soot predominate. The particles tend to be more mature, as the nanostructure characteristics show. At HAB = 40 mm, the average peak particle diameter of soot in O0, O5, and O10 flames are 15.8 nm, 16.1 nm, and 16.3 nm, respectively. The average particle size increases when the ozone concentration in the flame increases, and the surface growth of soot is stronger in ozone flames. The variation trend of the primary particle diameter in ethylene inverse diffusion flames is constant with the previous work [52] regardless of whether ozone is added.

### 3.3. Soot Surface Chemistry

**Figure 9:**
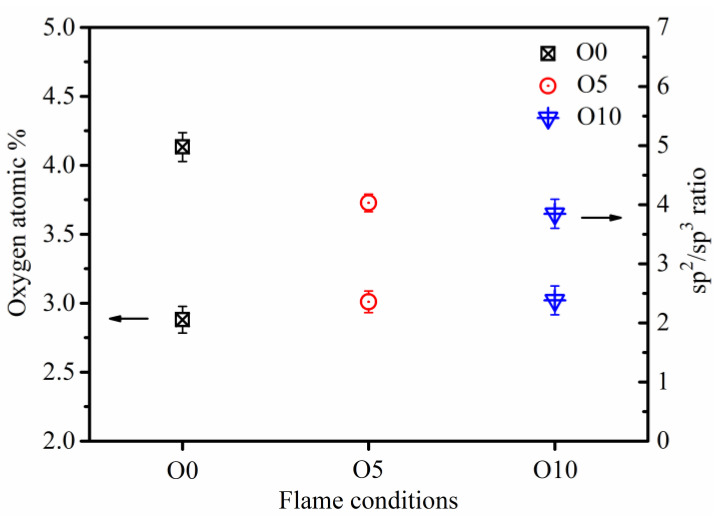
Surface oxygen content (left coordinate) and ratio of sp^2^/sp^3^ (right coordinate) of soot with different ozone concentrations.

The surface oxygen content of soot particles and the relative components of sp^2^ and sp^3^ carbon hybridization in the flames with different ozone concentrations are obtained by XPS quantitative analysis, as shown in Figure 9. The data at the lower position in the figure correspond to the left coordinate, representing the surface oxygen content of soot particles. The mean surface oxygen content of O0, O5, and O10 soot is 2.88%, 3.01%, and 3.02%, respectively. When the ozone concentration in the flame increases, the surface oxygen content of particles increases slightly, but the change is not obvious.

The XPS spectra of soot particles in flames with different ozone concentrations are fitted by peaking [53,54], and the ratio of sp^2^/sp^3^ obtained (data in the upper part of Figure 9) corresponds to the coordinate on the right of the figure. The sp^2^ content can indicate graphitic carbon, and the sp^3^ represents the content of the defect site and organic carbon [54,55]. Previous studies also indicated that oxygen content apparently affects the carbon chemical bonding state; particularly, the O-atom content of the combustion conditions alters the hybrid carbon component [54,56]. The actual role of the oxygen content in carbon hybridization is dependent on comprehensive factors including the nascent fuel composition, gas-phase pyrolysis processes, temperature history, etc. The ratio of sp^2^/sp^3^ can characterize the disorder degree of soot, and the smaller the ratio is, the more disordered the soot particles are. The ratio of sp^2^/sp^3^ of soot in O0, O5, and O10 flames is 4.97, 4.03, and 3.85, respectively. With the increase in ozone concentration, the ratio of sp^2^/sp^3^ decreases, which demonstrates that sp^3^ hybrid carbon content is higher in higher ozone concentration flames, indicating a lower degree of graphitization. This is because with higher ozone concentration in the flame, O_3_ decomposes to O_2_ and O radical, subsequently increasing the O-atom content in the flame and leading to an increase in the sp^3^ hybrid component. The soot nanostructure and disorder degree can affect soot oxidation reactivity [57].

### 3.4. Soot Oxidation Reactivity

**Figure 10:**
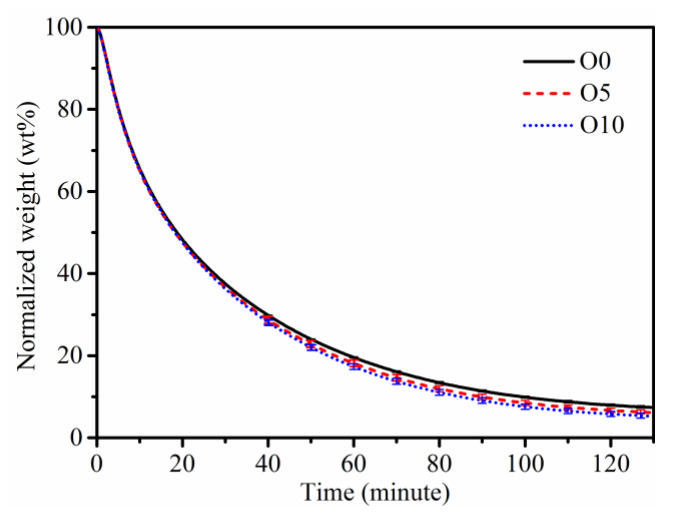
TGA results of soot at 500 °C from flames with different ozone concentrations.

Figure 10 shows the oxidation characteristic curves of soot obtained at different ozone concentrations during constant temperature oxidation at 500 °C. Since the difference in the ozone concentration is small, and considering the test error in the oxidation experiment, the oxidation curves with error range under different flame conditions are given in the figure. Within the allowable error range, the oxidation reactivity of soot generated in the O5 and O10 flame is very similar, and the oxidation reactivity in the O10 flame is slightly higher. The weight loss process of soot is slightly accelerated when the ozone concentration increases, indicating that the soot oxidation reactivity increases.

The average time from the initial oxidation of soot to 90% consumption is about 99.2, 89.8, and 84.9 min, respectively. According to the results of XPS analysis, the degree of graphitization decreases when ozone is added to the flame, which enhances the soot oxidation reactivity and makes it more easily oxidized. In addition, the content of volatile materials in the soot from the flame increases gradually with the increasing ozone content, corresponding to 11.5%, 13.2%, and 14.1% in O0, O5, and O10 flame, respectively. The higher volatile content on the soot surface with a higher ozone concentration leads to higher oxidation reactivity. The possible reason is that the decrease in volatile compounds increases the pore area on the particle surface, which facilitates contact with oxidants [58,59].

## 4. Conclusions

The soot formation and evolution profiles containing morphology and nanostructure, soot surface chemistry, and soot reactivity were studied in ethylene inverse diffusion flames with different ozone concentrations through a combination of the thermophoretic sampling method and quartz plate sampling method. Soot particles experienced inception, surface growth, agglomeration, and carbonization processes, moving from the bottom to the flame top in the ethylene inverse diffusion flames with or without ozone addition. The soot mass slightly increased under the ozone atmosphere. The soot formation and agglomeration were slightly advanced because the free radicals and active substances were promoted due to ozone decomposition. The diameter of primary particles was larger, and the degree of agglomeration of soot was higher in the ozone flames at the same sampling height. With the increase in ozone concentration in the flames, the soot surface oxygen content increased and the ratio of sp^2^/sp^3^ decreased. The soot generated from the flame with higher ozone concentration had higher disordered organization and a lower degree of graphitization, resulting in a higher oxidation reactivity.

## Figures and Tables

**Table 1:** Flame conditions.

Flame Notation	O_3_ Concentration (mg/L)	Gas Flow Rate (L/min)			
		C_2_H_4_	O_2_	N_2_ (Diluent)	N_2_ (Shield)
O0	0	0.45	0.25	0.45	13.0
O5	5.4 ± 0.3	0.45	0.25	0.45	13.0
O10	10.9 ± 0.5	0.45	0.25	0.45	13.0

## Data Availability

The data presented in this study are available on request from the corresponding author.

## References

[1] Liang X., Wang Z., Weng W., Zhou Z., Huang Z., Zhou J., Cen K. (2013). Study of ozone-enhanced combustion in H_2_/CO/N_2_/air premixed flames by laminar burning velocity measurements and kinetic modeling. Int. J. Hydrogen Energy.

[2] Baldelli A., Esmeryan K.D., Popovicheva O. (2021). Turning a negative into a positive: Trends, guidelines and challenges of developing multifunctional non-wettable coatings based on industrial soot wastes. Fuel.

[3] Jeyaseelan T., Ekambaram P., Subramanian J., Shamim T. (2022). A comprehensive review on the current trends, challenges and future prospects for sustainable mobility. Renew. Sustain. Energy Rev..

[4] Starikovskii A.Y. (2005). Plasma supported combustion. Proc. Combust. Inst..

[5] Karpenko E.I., Messerle V.E., Ustimenko A.B. (2007). Plasma-aided solid fuel combustion. Proc. Combust. Inst..

[6] Elaissi S., Ben Gouider Trabelsi A., Alkallas F.H., Alrebdi T.A., Charrada K. (2022). Modeling of advanced silicon nanomaterial synthesis approach: From reactive thermal plasma jet to nanosized particles. Nanomaterials.

[7] Kim W., Do H., Mungal M.G., Cappelli M.A. (2008). Optimal discharge placement in plasma-assisted combustion of a methane jet in cross flow. Combust. Flame.

[8] Leonov S.B., Savelkin K.V., Firsov A.A., Yarantsev D.A. (2010). Fuel ignition and flame front stabilization in supersonic flow using electric discharge. High Temp..

[9] Aleksandrov N.L., Kindysheva S.V., Kosarev I.N., Starikovskaia S.M., Starikovskii A.Y. (2009). Mechanism of ignition by non-equilibrium plasma. Proc. Combust. Inst..

[10] Smekhov G.D., Ibraguimova L.B., Karkach S.P., Skrebkov O.V., Shatalov O.P. (2007). Numerical simulation of ignition of a hydrogeneoxygen mixture in view of electronically excited components. High Temp..

[11] McClurkin J.D., Maier D.E. (2010). Half-life time of ozone as a function of air conditions and movement. Proceedings of the 10th International Working Conference on Stored Product Protection.

[12] Sun W., Gao X., Wu B., Ombrello T. (2019). The effect of ozone addition on combustion: Kinetics anddynamics. Proc. Combust. Inst..

[13] Nishida H., Tachibana T. (2006). Homogeneous charge compression ignition of natural gas/air mixture with ozone addition. J. Propuls. Power.

[14] Yamada H., Yoshii M., Tezaki A. (2005). Chemical mechanistic analysis of additive effects in homogeneous charge compression ignition of dimethyl ether. Proc. Combust. Inst..

[15] Halter F., Higelin P., Dagaut P. (2011). Experimental and detailed kinetic modeling study of the effect of ozone on the combustion of methane. Energy Fuels.

[16] Ombrello T., Won S.H., Ju Y., Williams S. (2010). Flame propagation enhancement by plasma excitation of oxygen. Part I: Effects of O_3_. Combust. Flame.

[17] Wang Z., Yang L., Li B., Li Z., Sun Z., Alden M., Cen K., Konnov A.A. (2012). Investigation of combustion enhancement by ozone additive in CH_4_/air flames using direct laminar burning velocity measurements and kinetic simulations. Combust. Flame.

[18] Gao X., Zhang Y., Adusumilli S., Seitzman J., Sun W., Ombrello T., Carter C. (2015). The effect of ozone addition on laminar flame speed. Combust. Flame.

[19] Foucher F., Higelin P., Mounaїm-Rousselle C., Dagaut P. (2013). Influence of ozone on the combustion of n-heptane in a HCCI engine. Proc. Combust. Inst..

[20] Masurier J., Foucher F., Dayma G., Dagaut P. (2015). Investigation of iso-octane combustion in a homogeneous charge compression ignition engine seeded by ozone, nitric oxide and nitrogen dioxide. Proc. Combust. Inst..

[21] Vu T.M., Won S.H., Ombrello T., Cha M.S. (2014). Stability enhancement of ozone-assisted laminar premixed Bunsen flames in nitrogen co-flow. Combust. Flame.

[22] Zhang Y., Zhu M., Zhang Z., Shang R., Zhang D. (2016). Ozone effect on the flammability limit and near-limit combustion of syngas/air flames with N_2_, CO_2_, and H_2_O dilutions. Fuel.

[23] Tachibana T., Hirata K., Nishida H., Osada H. (1991). Effect of ozone on combustion of compression ignition engines. Combust. Flame.

[24] Wilk M., Magdziarz A. (2010). Ozone effects on the emissions of pollutants coming from natural gas combustion. Polish J. Environ. Stud..

[25] Mok Y.S., Lee H.J. (2006). Removal of sulfur dioxide and nitrogen oxides by using ozone injection and absorption–reduction technique. Fuel Process. Technol..

[26] Wang Z., Zhou J., Zhu Y., Wen Z., Liu J., Cen K. (2007). Simultaneous removal of NOx, SO_2_ and Hg in nitrogen flow in a narrow reactor by ozone injection: Experimental results. Fuel Process. Technol..

[27] Sun W., Ding S., Zeng S., Sun S., Jiang W. (2011). Simultaneous absorption of NOx and SO_2_ from flue gas with pyrolusite slurry combined with gas-phase oxidation of NO using ozone. J. Hazard. Mater..

[28] Holder A.L., Carter B.J., Goth-Goldstein R., Lucas D., Koshland C.P. (2012). Increased cytotoxicity of oxidized flame soot. Atmos. Pollut. Res..

[29] Browne E.C., Franklin J.P., Canagaratna M.R., Massoli P., Kirchstetter T.W., Worsnop D.R., Wilson K.R., Kroll J.H. (2015). Changes to the chemical composition of soot from heterogeneous oxidation reactions. J. Phys. Chem. A.

[30] Daly H.M., Horn A.B. (2009). Heterogeneous chemistry of toluene, kerosene and diesel soots. Phys. Chem. Chem. Phys..

[31] Ying Y., Liu D. (2018). Nanostructure evolution and reactivity of nascent soot from inverse diffusion flames in CO_2_, N_2_, and He atmospheres. Carbon.

[32] Ying Y., Liu D. (2021). Soot properties in ethylene inverse diffusion flames blended with different carbon chain length alcohols. Fuel.

[33] Ying Y., Liu D. (2019). Effects of water addition on soot properties in ethylene inverse diffusion flames. Fuel.

[34] Ying Y., Liu D. (2017). Effects of butanol isomers additions on soot nanostructure and reactivity in normal and inverse ethylene diffusion flames. Fuel.

[35] McEnally C.S., Köylü Ü.Ö., Pfefferle L.D., Rosner D.E. (1997). Soot volume fraction and temperature measurements in laminar nonpremixed flames using thermocouples. Combust. Flame.

[36] Merchan-Merchan W., Abdihamzehkolaei A., Merchan-Breuer D.A. (2018). Formation and evolution of carbon particles in coflow diffusion air flames of vaporized biodiesel, diesel and biodiesel-diesel blends. Fuel.

[37] Abid A.D., Heinz N., Tolmachoff E.D., Phares D.J., Campbell C.S., Wang H. (2008). On evolution of particle size distribution functions of incipient soot in premixed ethylene-oxygen-argon flames. Combust. Flame.

[38] Dobbins R.A., Fletcher R.A., Chang H.C. (1998). The evolution of soot precursor particles in a diffusion flame. Combust. Flame.

[39] Wang H. (2011). Formation of nascent soot and other condensed-phase materials in flames. Proc. Combust. Inst..

[40] Blevins L.G., Fletcher R.A., Benner B.A., Steel E.B., Mulholland G.W. (2002). The existence of young soot in the exhaust of inverse diffusion flames. Proc. Combust. Inst..

[41] D’Anna A. (2009). Combustion-formed nanoparticles. Proc. Combust. Inst..

[42] Kholghy M., Saffaripour M., Yip C., Thomson M.J. (2013). The evolution of soot morphology in a laminar coflow diffusion flame of a surrogate for Jet A-1. Combust. Flame.

[43] De Falco G., Sirignano M., Commodo M., Merotto L., Migliorini F., Dondè R., De Iuliis S., Minutolo P., D’Anna A. (2018). Experimental and numerical study of soot formation and evolution in coflow laminar partially premixed flames. Fuel.

[44] Desgroux P., Mercier X., Thomson K.A. (2013). Study of the formation of soot and its precursors in flames using optical diagnostics. Proc. Combust. Inst..

[45] Velásquez M., Mondragón F., Santamaría A. (2013). Chemical characterization of soot precursors and soot particles produced in hexane and diesel surrogates using an inverse diffusion flame burner. Fuel.

[46] Alfè M., Apicella B., Barbella R., Rouzaud J.N., Tregrossi A., Ciajolo A. (2009). Structure-property relationship in nanostructures of young and mature soot in premixed flames. Proc. Combust. Inst..

[47] Won S.H., Jiang B., Diévart P., Sohn C.H., Ju Y. (2015). Self-sustaining n-heptane cool diffusion flames activated by ozone. Proc. Combust. Inst..

[48] Frenklach M. (2002). Reaction mechanism of soot formation in flames. Phys. Chem. Chem. Phys..

[49] Frenklach M., Mebel A.M. (2020). On the mechanism of soot nucleation. Phys. Chem. Chem. Phys..

[50] Martin J.W., Salamanca M., Kraft M. (2022). Soot inception: Carbonaceous nanoparticle formation in flames. Prog. Energy Combust. Sci..

[51] Oh K.C., Lee U.D., Shin H.D., Lee E.J. (2005). The evolution of incipient soot particles in an inverse diffusion flame of ethene. Combust. Flame.

[52] Naseri A., Veshkini A., Thomson M.J. (2017). Detailed modeling of CO_2_ addition effects on the evolution of soot particle size distribution functions in premixed laminar ethylene flames. Combust. Flame.

[53] Vander Wal R.L., Bryg V.M., Huang C.-H. (2014). Aircraft engine particulate matter: Macro- micro- and nanostructure by HRTEM and chemistry by XPS. Combust. Flame.

[54] Vander Wal R.L., Bryg V.M., Hays M.D. (2011). XPS analysis of combustion aerosols for chemical composition, surface chemistry, and carbon chemical state. Anal. Chem..

[55] Pumera M., Iwai H. (2009). Multicomponent metallic impurities and their influence upon the electrochemistry of carbon nanotubes. J. Phys. Chem. C.

[56] Esmeryan K.D., Castano C.E., Bressler A.H., Abolghasemibizaki M., Mohammadi R. (2016). Rapid synthesis of inherently robust and stable superhydrophobic carbon soot coatings. Appl. Surf. Sci..

[57] Song J., Alam M., Boehman A.L. (2007). Impact of alternative fuels on soot properties and DPF regeneration. Combust. Sci. Technol..

[58] Yehliu K., Vander Wal R.L., Armas O., Boehman A.L. (2012). Impact of fuel formulation on the nanostructure and reactivity of diesel soot. Combust. Flame.

[59] Collura S., Chaoui N., Azambre B., Finqueneisel G., Heintz O., Krzton A., Weber J.V. (2005). Influence of the soluble organic fraction on the thermal behaviour, texture and surface chemistry of diesel exhaust soot. Carbon.

